# Effectiveness of acupuncture on pregnancy outcomes in patients with repeated implantation failure after IVF-ET: study protocol for a pilot randomized controlled trial

**DOI:** 10.3389/fendo.2026.1716274

**Published:** 2026-05-11

**Authors:** Ruiwen Fan, Yang Ye, Kun Liu, Jing Xu, Yutian Zhu, Haolin Zhang, Yu Gao, Xiyan Xin, Dong Li

**Affiliations:** State Key Laboratory of Female Fertility Promotion, Department of Traditional Chinese Medicine, Peking University Third Hospital, Beijing, China

**Keywords:** acupuncture, embryo implantation rate, *in vitro* fertilization-embryo transfer, randomized controlled trial, repeated implantation failure

## Abstract

**Objective:**

Repeated implantation failure (RIF) in *in vitro* fertilization-embryo transfer (IVF-ET) remains a challenging clinical issue. Although acupuncture is increasingly used as an adjunctive therapy for RIF patients, robust evidence supporting its efficacy in improving pregnancy outcomes is still lacking. This pilot study therefore aims to estimate effect sizes to inform sample size calculations for future definitive trials and to assess key feasibility parameters.

**Design and methods:**

This single-center, two-arm, single-blind, randomized sham-controlled trial will enroll 100 women (50 per group) with RIF aged 25~40 years. Using a parallel-group design, participants will be randomized to receive either conventional Western medicine treatment combined with acupuncture (experimental group) or the same conventional Western medicine treatment combined with sham acupuncture (control group). Acupuncture or sham acupuncture will be administered twice weekly from day 5 of the menstrual cycle until the day before embryo transfer, with a minimum of three completed sessions, targeting acupoints with standardized techniques. The primary outcome will be embryo implantation rate, and secondary outcomes will include clinical pregnancy rate and serum biomarkers (β-human chorionic gonadotropin, luteinizing hormone, progesterone, and estradiol). Safety will be monitored through adverse event reporting.

**Discussion:**

The findings of this study will provide preliminary data to guide the design of future large-scale, multicenter randomized controlled trials. Such trials would in turn generate more definitive evidence on the efficacy of acupuncture for improving pregnancy outcomes in RIF patients undergoing IVF-ET.

**Clinical trial registration:**

https://itmctr.ccebtcm.org.cn/, identifier ITMCTR2025000838

## Introduction

*In vitro* fertilization-embryo transfer (IVF-ET), as a core technique of assisted reproductive technology (ART), has helped millions of infertile patients worldwide fulfill their desire to have children. However, recurrent implantation failure (RIF), one of the major bottlenecks in IVF-ET treatment, affects approximately 10% to 15% of the patient population (1). RIF is defined as the failure to achieve a clinical pregnancy after consecutive transfers of ≥2 high-quality embryos (2). It not only imposes significant economic and psychological burdens but also potentially undermines patients’ confidence in the treatment (3). Despite the fact that recent advancements in embryo screening technologies (4) and endometrial receptivity testing (5) have notably improved the synchrony between embryos and the endometrium, the pathological mechanisms underlying RIF remain highly heterogeneous, involving multiple factors such as imbalances in the endometrial microenvironment (6), dysregulation of immune modulation at the maternal-fetal interface (7), and insufficient blood perfusion in the uterine spiral arteries (8). This complexity necessitates multi-target regulatory approaches in clinical intervention strategies for RIF.

In this context, acupuncture therapy, rooted in the holistic view and syndrome differentiation and treatment theory of traditional Chinese medicine (TCM), has gradually attracted attention. Modern research indicates that acupuncture may improve the reproductive microenvironment through multiple pathways. Acupuncture is hypothesized to optimize estrogen and progesterone secretion by regulating the hypothalamic-pituitary-ovarian axis, thus potentially prolonging endometrial receptivity (9). It may also increases uterine artery perfusion and resistance indices, thereby potentially promoting endometrial angiogenesis (10). Moreover, its proposed regulation of immune balance offers a plausible mechanism to improve embryo implantation (11). Previous clinical studies have suggested that acupuncture as an adjunct to IVF-ET may increase the clinical pregnancy rate and live birth rate in patients with RIF, and the reported improvement in embryo implantation rate has been statistically significant (12–14).

Based on this, we designed a prospective, randomized, controlled pilot trial comparing short-term acupuncture versus sham acupuncture—both combined with conventional Western medicine—in women with RIF undergoing IVF-ET. The trial employed a modified Streitberger non-invasive placebo needle as the sham acupuncture control. The primary endpoint was embryo implantation rate, with secondary endpoints including clinical pregnancy rate and changes in reproductive hormone levels. By adhering to international reporting standards, this study aims to provide relatively high-level preliminary evidence for acupuncture in RIF treatment while informing the design of future multicenter trials. If validated in subsequent confirmatory studies, this low-cost, low-risk intervention could serve as a valuable addition to comprehensive RIF management protocols.

## Methods

### Study design

**Figure 1:**
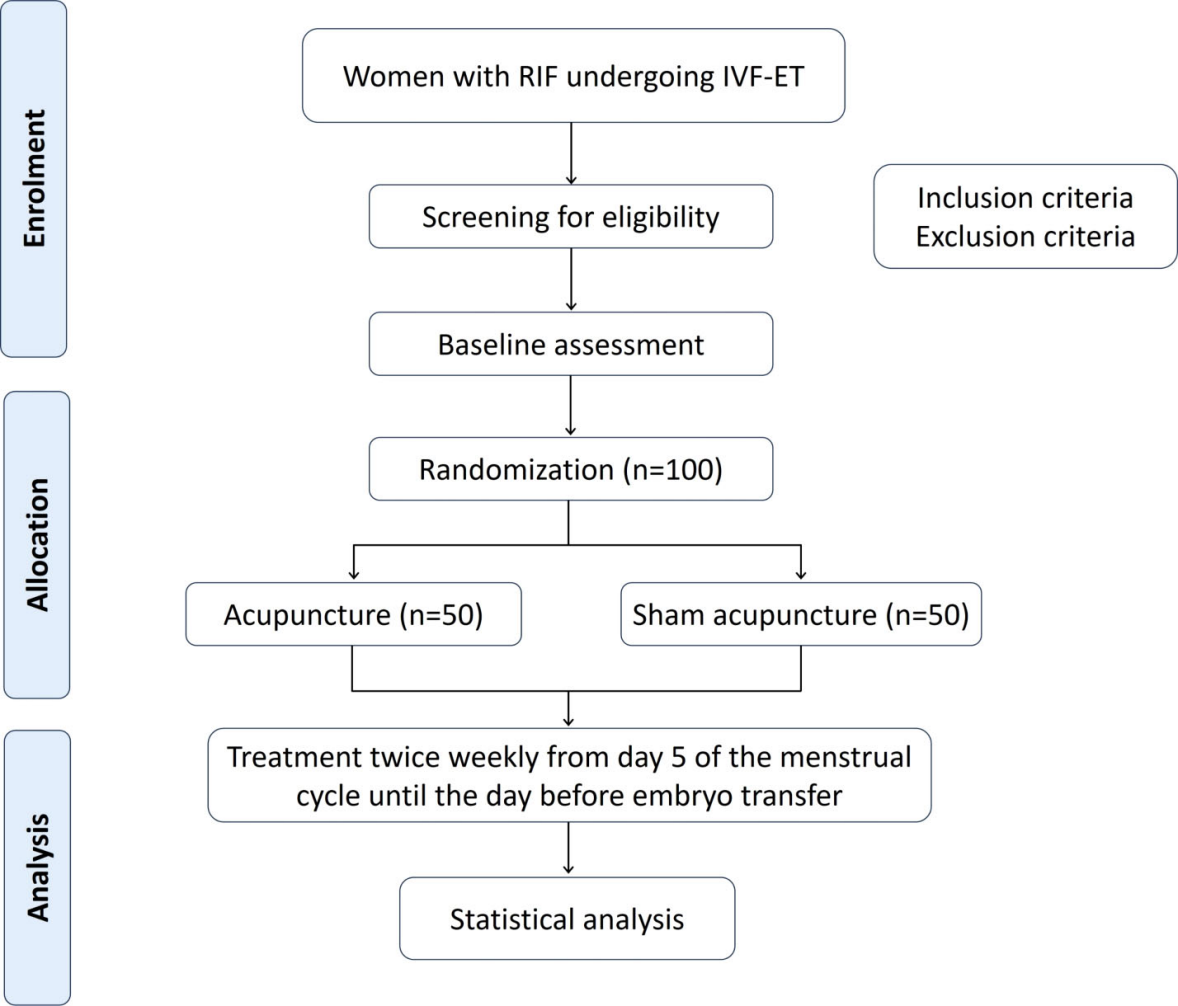
Study flow diagram. IVF-ET, *in vitro* fertilization-embryo transfer; RIF, repeated implantation failure.

This single-center, parallel-group, randomized, placebo-controlled trial will employ a patient-blinded design and be conducted at Peking University Third Hospital between December 2024 and December 2026. A total of 100 eligible participants will be enrolled and randomly allocated in a 1:1 ratio to either the experimental or control group. The trial flow is depicted in **Figure 1**. The study protocol adheres to the CONSORT (Consolidated Standards of Reporting Trials) (15) and STRICTA (Standards for Reporting Interventions in Clinical Trials of Acupuncture) (16) guidelines to ensure methodological rigor. Ethical approval has been obtained, and the trial was registered on October 21, 2024 (Registration number: ITMCTR2025000838).

### Ethical considerations

All participants will provide written informed consent before randomization. Written informed consent will be obtained directly from the participants themselves. The study protocol has been approved by the ethics committees of Peking University Third Hospital (Approval number: M2024352). Findings from this trial will be submitted for publication in peer-reviewed journals following completion. Patient confidentiality will be strictly maintained, with all data anonymized and securely stored. Participants may withdraw from the study at any time without penalty.

### Participants

This study will enroll patients diagnosed with RIF who are scheduled to undergo IVF-ET at Peking University Third Hospital between December 2024 and December 2026. All participants will receive standard baseline treatment as part of routine clinical care. Potential participants expressing interest in the trial will be contacted by the research team for preliminary screening. During the initial contact, researchers will document baseline demographic and clinical characteristics, perform a comprehensive eligibility assessment using predetermined inclusion/exclusion criteria, and provide detailed study information to ensure participants can make fully informed decisions.

### Inclusion criteria

1. Females aged 25–40 years.
2. Meeting the WHO diagnostic criteria for infertility (failure to achieve clinical pregnancy after ≥12 months of regular unprotected intercourse).
3. History of ≥2 unsuccessful IVF-ET cycles with availability of ≥1 good-quality cryopreserved embryo.
4. Normal ovarian reserve ((AMH ≥1.1 ng/mL or AFC ≥5) and regular menstrual cycles (21–35 days).
5. Willingness to participate as evidenced by signed informed consent document.

### Exclusion criteria

1. Patients with active genital tract infections or systemic infections requiring treatment.
2. Patients with significant congenital or acquired reproductive organ malformations or severe organic lesions.
3. Patients with endocrine disorders that may affect implantation, including: (a) untreated or poorly controlled thyroid dysfunction (defined as TSH < 0.3 mIU/L or > 4.5 mIU/L); (b) hyperprolactinemia (serum prolactin > 25 ng/mL); (c) polycystic ovary syndrome (PCOS) diagnosed according to Rotterdam criteria; (d) luteal phase defect (serum progesterone < 10 ng/mL on cycle day 21).
4. Patients with immunological or hematological disorders that may affect implantation, including: (a) autoimmune diseases; (b) inherited or acquired thrombophilia.
5. Patients exhibiting inadequate endometrial development (thickness <6 mm or Type C pattern) on trigger day.
6. Patients with a history of TORCH infection.
7. Patients with a Body Mass Index (BMI) <18.5 kg/m².

### Randomization and blinding

A total of 100 eligible participants will be randomly allocated to either the experimental or control group in a 1:1 ratio using permuted block randomization. The allocation sequence will be concealed through sequentially numbered, opaque, sealed envelopes to ensure randomization integrity. This trial employs a single-blind design wherein participants will remain blinded to their group assignment throughout the trial. Practitioners administering acupuncture will necessarily be unblinded due to the inherent characteristics of the intervention. To minimize detection bias, outcome assessors (including ultrasound technicians evaluating gestational sacs and laboratory personnel analyzing hormone levels) will be strictly segregated from the acupuncture procedures and will remain blinded to group allocation. Furthermore, the statistician responsible for data analysis will also be blinded until the analysis plan is finalized and the database is locked. While performance bias cannot be entirely eliminated due to practitioner unblinding, the use of a standardized sham protocol aims to mitigate differential participant-provider interactions.

### Procedure

All participants will receive standard Western medical treatment during their IVF-ET cycles, including ovulation induction medications in the preparatory phase and luteal phase support following embryo transfer. The administration of these foundational medications cannot be fully standardized due to interpatient variability in physiological parameters. Treatment protocols are therefore individualized based on patient characteristics to maximize pregnancy success rates. To address potential confounding, the following IVF cycle characteristics will be prospectively documented for each participant: specific controlled ovarian stimulation protocol (e.g., GnRH agonist long protocol, GnRH antagonist protocol, or others), type and dosage of gonadotropins, embryo stage at transfer (cleavage stage or blastocyst), embryo quality grade, and number of embryos transferred. Acupuncture or sham acupuncture intervention will be administered twice weekly from day 5 of the menstrual cycle until the day before embryo transfer, with a minimum requirement of three completed acupuncture sessions.

### Acupuncture treatment

**Figure 2:**
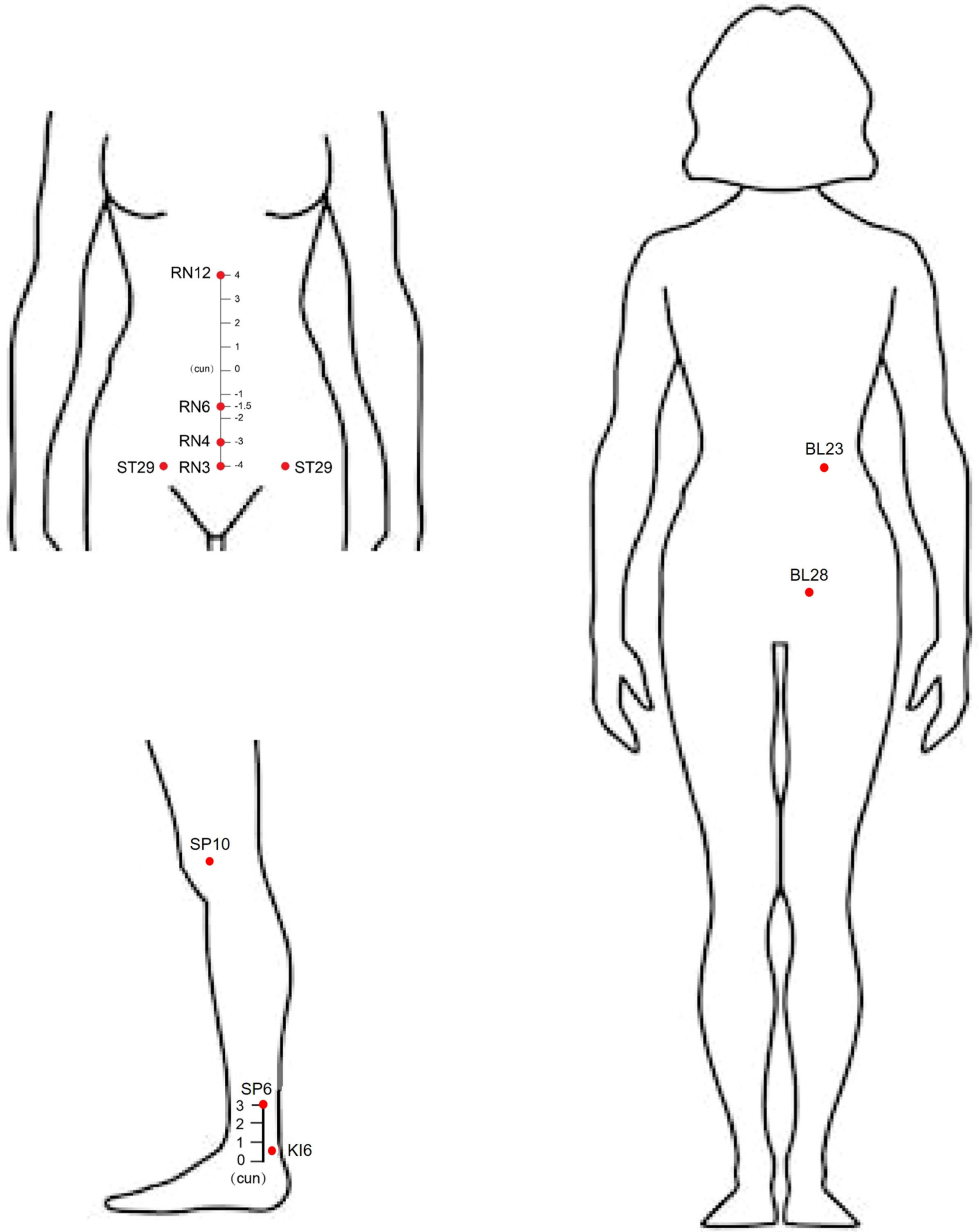
Locations of acupoints.

In accordance with TCM meridian theory, licensed acupuncturists will identify and mark the following acupoints: *Zhongwan* (RN12), *Qihai* (RN6), *Guanyuan* (RN4), *Zhongji* (RN3), bilateral *Guilai* (ST29), bilateral *Shenshu* (BL23), bilateral *Ciliao* (BL28), bilateral *Xuehai* (SP10), bilateral *Sanyinjiao* (SP6), and bilateral *Taixi* (KI6), with their precise anatomical locations detailed in **Table 1** and illustrated in **Figure 2**. Following standard aseptic protocol, all acupoints will be disinfected with 75% medical alcohol before inserting single-use sterile acupuncture needles (0.3×40 mm), then applying manual stimulation (twirling, lifting, and thrusting techniques) to elicit *de qi* sensation (characterized by soreness, numbness, distension, or heaviness), while four abdominal acupoints (*Zhongwan, Qihai, Guanyuan*, and *Zhongji*) will receive additional electroacupuncture stimulation using a G6805-1A device set at 2 Hz low-frequency continuous wave, with each acupuncture session lasting 30 minutes.

**Table 1:** Location of acupoints used.

Acupoints	Locations	Needling method
*Zhongwan* (RN12)	Midline, 4 cun above the umbilicus	Straight insertion 1–1.2 cun, EA
*Qihai* (RN6)	Midline, 1.5 cun below the umbilicus	Straight insertion 1–1.2 cun, EA
*Guanyuan* (RN4)	Midline, 3 cun below the umbilicus	Straight insertion 0.8–1.2 cun, EA
*Zhongji* (RN3)	Midline, 4 cun below the umbilicus	Straight insertion 0.8–1.0 cun, EA
*Guilai* (ST29)	2 cun lateral to RN4, lower abdomen	Straight insertion 0.8–1.0 cun
*Shenshu* (BL23)	1.5 cun lateral to the spinous process of L2	Straight insertion 0.8–1.0 cun
*Ciliao* (BL28)	Sacral foramen, level with the 2nd sacral vertebra	Straight insertion 1–1.2 cun
*Xuehai* (SP10)	Medial aspect of the thigh, 2 cun above the superior border of the patella	Straight insertion 1–1.5 cun
*Sanyinjiao* (SP6)	Medial leg, 3 cun above the medial malleolus	Straight insertion 1–1.2 cun
*Taixi* (KI6)	Medial ankle, between the tendons of Achilles and flexor digitorum longus	Straight insertion 0.8–1.2 cun

EA, electroacupuncture.

### Sham acupuncture treatment

This trial will employ sham acupuncture as the control to verify the efficacy of acupuncture. A non-penetrating sham acupuncture protocol will be implemented using modified Streitberger placebo needles (17). During the procedure, the placebo needle will be secured to the skin at corresponding acupoints through a plastic ring. Patients will experience a pricking sensation that mimics skin penetration, while in reality, the needle tip will retract into the sleeve without piercing the skin. The sham acupuncture group will receive stimulation at the same acupoints (*Zhongwan, Qihai, Guanyuan*, and *Zhongji*) as the verum acupuncture group. Additionally, electrodes from the electroacupuncture device will be attached to these acupoints in the sham acupuncture group to maintain procedural consistency, though no electrical current will be delivered. To maintain blinding integrity, the electroacupuncture device will be activated with indicator lights illuminated and the machine will produce an audible hum, mimicking the operational state of the active treatment group.

### Practitioner qualification and standardization

All acupuncture procedures will be performed by licensed acupuncturists with a minimum of five years of clinical experience in reproductive medicine. Prior to trial initiation, all practitioners will complete a standardized training session covering the precise anatomical location of each acupoint, needle manipulation techniques to achieve *de qi*, and the operation of the electroacupuncture device. Mechanistic rationale for acupoint selection: The selected acupoints are based on both TCM theory and modern research. Zhongwan (RN12), Qihai (RN6), Guanyuan (RN4), and Zhongji (RN3) are located on the Ren Meridian and are traditionally used to regulate the uterus and nourish the Chong and Ren vessels, which govern reproduction. Guilai (ST29) and Shenshu (BL23) tonify the kidney and regulate menstruation. Ciliao (BL28) targets the sacral parasympathetic nerves, potentially enhancing uterine blood flow. Xuehai (SP10), Sanyinjiao (SP6), and Taixi (KI6) are used to activate blood circulation, resolve stasis, and balance endocrine function.

### Outcomes

#### Primary outcomes

The primary outcome is embryo implantation rate, defined as the number of implanted embryos per transferred embryo (18).

#### Secondary outcomes

1. Clinical pregnancy rate. Presence of a gestational sac with embryonic cardiac activity confirmed by transvaginal ultrasound at 30 days post-embryo transfer (19).

2. Hormonal profiles. Serum levels of β-human chorionic gonadotropin (β-hCG), luteinizing hormone (LH), progesterone (P), and estradiol (E2) measured at 14 days post-transfer.

### Safety evaluation

Adverse events (e.g., needle fainting, hematoma, or drug-related adverse reactions) will be systematically documented and severity-graded in accordance with the Common Terminology Criteria for Adverse Events (CTCAE) version 5.0 (20).

### Data management

Trained researchers will record all data using standardized case report forms (CRFs). To ensure data accuracy, we will employ a dual-entry system with automated consistency validation, with any discrepancies adjudicated by the principal investigators.

### Quality control

An expert panel comprising specialists in acupuncture, obstetrics/gynecology, methodology, and biostatistics has rigorously reviewed the trial protocol. To minimize bias, we will implement centralized randomization. Prior to trial initiation, all research staff will undergo standardized training covering: 1) participant recruitment procedures, 2) randomization protocols, 3) acupuncture intervention techniques, and 4) patient communication strategies. Data will be systematically recorded using uniform CRFs, with monthly trial monitoring conducted by the research team.

### Sample size

Based on previously reported data, the embryo implantation rate in RIF patients receiving acupuncture combined with hormone replacement therapy (HRT) is estimated at 35.42%, compared with 20.13% in patients receiving HRT alone (21). To detect this difference with a two-sided significance level of α = 0.05 and a power of 90% (1-β = 0.90) using a 1:1 allocation ratio, a total of 358 participants (179 per group) would be required for a fully powered confirmatory randomized controlled trial (calculated using the formula for comparing two independent proportions with continuity correction).

As an exploratory clinical study designed with pragmatic considerations, we determined the sample size through comprehensive evaluation of feasibility constraints rather than formal power calculations. This pragmatic approach is consistent with the methodological guidance for pilot trials, which emphasizes that sample sizes should be based on practical considerations. The final sample of 100 participants (50 per group) has been established after accounting for anticipated recruitment challenges, 24-month study duration limitations, available research funding, and current staffing capacity. We acknowledge that with a sample size of 100, the trial may have limited statistical power to detect small-to-moderate treatment effects. Therefore, the trial is not designed or powered to produce definitive efficacy conclusions, and the results will be interpreted with appropriate caution. The findings will primarily serve to generate preliminary estimates of between-group differences in embryo implantation rate, inform formal power calculations for a subsequent large-scale, multicenter RCT, and evaluate protocol feasibility parameters including recruitment rate, retention rate, and protocol adherence.

### Statistical analysis

Baseline characteristics will be summarized using descriptive statistics, with continuous variables reported as mean ± standard deviation or median (interquartile range) depending on data distribution, and categorical variables reported as frequencies and percentages. The following baseline characteristics will be collected and compared between groups: age, BMI, duration of infertility, number of previous failed IVF-ET cycles, basal FSH level, AMH level, AFC, endometrial thickness, and number of embryos transferred. Between-group comparisons of baseline characteristics will employ independent samples t-tests or Mann-Whitney U tests for continuous variables, and χ² tests or Fisher’s exact tests for categorical variables, as appropriate.

Before conducting comparative analysis tests on all variables, a data normality test will be performed first to ensure the appropriateness of the selected comparative analysis methods. For the primary outcome, embryo implantation rate, the unit of analysis is defined as the individual embryo. However, because each participant may contribute multiple embryos, the data exhibit a clustered structure that violates the independence assumption of conventional statistical tests. Therefore, between-group comparisons for the primary outcome will be performed using a Generalized Estimating Equation (GEE) model with a logit link function and a binomial distribution family. An exchangeable correlation structure will be specified to account for within-patient clustering. The model will include treatment group (acupuncture vs. sham acupuncture) as the primary independent variable, and will be adjusted for the following pre-specified clinically relevant covariates: age, BMI, number of previous failed IVF-ET cycles, controlled ovarian stimulation protocol type (categorical), embryo stage (cleavage vs. blastocyst), embryo quality (good vs. fair, dichotomized for analysis), and number of embryos transferred. Results will be reported as odds ratios with 95% confidence intervals and P-values based on robust standard errors. For secondary outcomes, between-group comparisons will employ: 1) χ² or Fisher’s exact tests for dichotomous outcomes (clinical pregnancy rate); 2) independent samples t-tests or Mann-Whitney U tests for continuous variables (reproductive hormone levels). The intention-to-treat population will serve as the primary analysis set, with missing outcome data will be handled using multiple imputation by chained equations (MICE) under the assumption of missing at random (MAR), with sensitivity analyses performed under missing not at random (MNAR) assumptions to assess the robustness of the findings.

In addition to the primary analysis, sensitivity analyses will be performed to assess the robustness of the primary outcome, including per-protocol analysis and complete-case analysis. Exploratory subgroup analyses will be conducted to generate hypotheses for future research. These analyses will evaluate the primary outcome stratified by age (25-30, 30-35, or 35–40 years), BMI categories (normal weight: 18.5≤BMI<23; overweight: 23≤BMI< 27.5; obese: BMI≥27.5) (22), and the number of acupuncture sessions received (3, 4, 5, or ≥6 sessions). Given the limited sample size of this pilot trial, these subgroup analyses are purely descriptive and will be presented without formal statistical comparisons between subgroups to avoid spurious findings. No adjustment for multiple testing will be applied, as the purpose is hypothesis generation rather than confirmatory inference. The results will be interpreted with extreme caution and will serve solely to inform the design of future larger trials. All analyses will be performed using SPSS software, with statistical significance defined as a two-tailed *P*-value <0.05.

## Discussion

RIF represents a significant challenge in IVF-ET. Although preliminary evidence suggests that acupuncture may improve pregnancy outcomes in RIF patients, the current evidence base is limited by methodological weaknesses. This exploratory pilot randomized controlled trial is therefore designed to evaluate the effects of acupuncture on pregnancy outcomes in women with RIF undergoing IVF-ET, with a focus on estimating effect sizes and assessing feasibility. The findings will provide foundational data to inform the design of future large-scale, multicenter confirmatory RCTs.

This study employs an acupuncture protocol, which is significantly more condensed than conventional approaches. The treatment will be administered twice weekly, starting on day 5 of the menstrual cycle and continuing until the day before embryo transfer. Assuming a typical 28-day cycle with ovulation occurring around day 14, this results in approximately 13 days of treatment (though the exact duration may vary depending on individual cycle length and embryo transfer scheduling). Developed based on our clinical experience, this protocol differs markedly from traditional RIF acupuncture regimens that often require months of treatment (12). By compressing the intervention period to just two weeks, our approach substantially reduces patient treatment duration while maintaining therapeutic feasibility. We acknowledge that this condensed protocol may deliver a lower cumulative acupuncture dose than conventional long−term regimens, which could potentially increase the risk of a false−negative finding. However, as a pilot trial, establishing feasibility of a pragmatic, time−efficient protocol is a primary objective, and the effect size estimates generated will inform dose optimization in future studies.

To rigorously evaluate the efficacy of acupuncture for RIF, this study employs sham acupuncture combined with conventional Western medicine treatment as the control group. Sham acupuncture is widely accepted in acupuncture research as a control intervention, as it effectively minimizes placebo effects associated with needle insertion (23). The selection of a sham acupuncture control rather than a usual care control reflects the explanatory nature of this trial. While a pragmatic design comparing acupuncture plus conventional treatment to conventional treatment alone might better reflect real-world practice, the current pilot study aims to isolate the specific effect of acupuncture needling from non-specific placebo effects. The use of a validated non-penetrating sham device allows for participant blinding and provides a rigorous test of the acupuncture intervention’s efficacy.

Regarding blinding, only participants will be blinded in this trial. Practitioners cannot be blinded due to the necessity of performing the acupuncture procedures (24). This study does not include long-term follow-up assessments. This design decision is based on two key considerations: 1) the transient nature of acupuncture effects is unlikely to persist for extended periods, and 2) post-treatment follow-up conducted months later would not accurately reflect the actual intervention effects. The immediate post-treatment evaluation window was therefore determined to be most appropriate for capturing the therapeutic impact of our acupuncture protocol.

The primary outcome of this study is embryo implantation rate, as our primary focus is determining whether IVF-ET successfully achieves embryo implantation. This selection is based on the mechanistic focus and practical constraints of this pilot trial. First, the study examines whether brief acupuncture during the peri−implantation window may directly influence embryo attachment through potential enhancement of endometrial receptivity and local blood flow. Second, limited follow−up time and funding make it infeasible to track participants to live birth, which requires around 9 months of monitoring after transfer. Third, although clinical pregnancy and live birth rates are more clinically meaningful, they are affected by many post−implantation factors unrelated to acupuncture’s mechanism. In a small sample, these factors would dilute the intervention effect and reduce the reliability of estimated effect sizes. Thus, implantation rate is a direct and biologically appropriate surrogate that optimizes the signal−to−noise ratio in this exploratory study. For secondary outcomes, we have chosen clinical pregnancy rate and reproductive hormone levels. As quantifiable objective measures, reproductive hormones serve as important biomarkers influencing pregnancy outcomes and can help elucidate the potential mechanisms through which the intervention may exert its effects. Nevertheless, we acknowledge that embryo implantation rate is a surrogate endpoint rather than a definitive clinical outcome. Consequently, the findings of this pilot trial are not intended to directly guide clinical decision−making, but rather to inform the design and power calculations of future confirmatory trials with more clinically meaningful endpoints such as live birth rate.

Considering the prevalent treatment anxiety, time constraints and high concern for pregnancy outcomes among IVF patients, this protocol was designed with a strategy combining individualization and standardization. Acupuncture was delivered at critical time points during the IVF cycle, with each session lasting 30 minutes and a frequency of twice weekly, to avoid extra psychological and time burdens. Taking into account the complexity of clinical workflows in real−world IVF settings, the acupuncture treatment was provided in a TCM room inside the reproductive center, enabling a one−stop service immediately after routine procedures such as ultrasound monitoring without repeated travel between departments. A strict adverse event recording system was implemented. For mild adverse events such as slight subcutaneous bruising, verbal education with scientific explanations and cold compress guidance was offered to relieve patients’ unnecessary fear. This protocol is highly compatible with routine IVF clinical pathways, and patients generally rated the treatment schedule as flexible and non−disruptive, indicating excellent treatment acceptability.

This proposed trial has several limitations that should be acknowledged. First, the absence of a formal sample size calculation means the study may be underpowered to detect statistically significant differences. Second, while participants and outcome assessors are blinded, practitioners are necessarily unblinded, which introduces a potential for performance bias. Moreover, although the sham device mimics needle insertion, the sensation of *de qi* in the verum acupuncture group or the perception of electrical stimulation may lead to partial unblinding of participants. As a pilot trial, we did not include a formal blinding credibility assessment to avoid prompting additional scrutiny that could itself bias outcomes. We acknowledge that the absence of a formal blinding evaluation represents a methodological limitation of this pilot study. To address this, future definitive trials will incorporate a validated blinding credibility questionnaire administered immediately following the intervention period to formally quantify the success of participant blinding. Third, as a single-center study, the generalizability of our findings to broader populations remains uncertain and warrants further investigation in multicenter trials. Furthermore, the non−penetrating sham acupuncture device, while effective for blinding, may not be entirely physiologically inert, as pressure and tactile stimulation at acupoints could theoretically produce mild therapeutic effects. This limitation is inherent to most sham acupuncture controls and may bias effect size estimates toward the null, reducing the observed between−group difference. Additionally, the use of a standardized acupoint prescription without TCM syndrome differentiation, while essential for ensuring internal validity and replicability in a randomized trial, may not fully reflect the individualized approach employed in real−world clinical practice. The generalizability of our findings to personalized acupuncture protocols therefore warrants cautious interpretation.
